# Evaluation of the vaginal flora in pregnant women receiving opioid maintenance therapy: a matched case-control study

**DOI:** 10.1186/s12884-016-1003-z

**Published:** 2016-08-05

**Authors:** Alex Farr, Herbert Kiss, Michael Hagmann, Iris Holzer, Verena Kueronya, Peter W. Husslein, Ljubomir Petricevic

**Affiliations:** 1 Department of Obstetrics and Gynaecology, Division of Obstetrics and Fetomaternal Medicine, Medical University of Vienna, Waehringer Guertel 18-20, A-1090 Vienna, Austria; 2 Section for Medical Statistics, Centre of Medical Statistics, Informatics and Intelligent Systems, Medical University of Vienna, Vienna, Austria

**Keywords:** Bacterial vaginosis, *Candida*, Infection, Opioid addiction, Pregnancy, Preterm delivery

## Abstract

**Background:**

Vaginal infections are a risk factor for preterm delivery. In this study, we sought to evaluate the vaginal flora of pregnant women receiving opioid maintenance therapy (OMT) in comparison to non-dependent, non-maintained controls.

**Methods:**

A total of 3763 women with singleton pregnancies who underwent routine screening for asymptomatic vaginal infections between 10 + 0 and 16 + 0 gestational weeks were examined. Vaginal smears were Gram-stained, and microscopically evaluated for bacterial vaginosis, candidiasis, and trichomoniasis. In a retrospective manner, data of 132 women receiving OMT (cases) were matched for age, ethnicity, parity, education, previous preterm delivery, and smoking status to the data of 3631 controls. The vaginal flora at antenatal screening served as the primary outcome measure. Secondary outcome measures were gestational age and birth weight.

**Results:**

In the OMT group, 62/132 (47 %) pregnant women received methadone, 39/132 (29.5 %) buprenorphine, and 31/132 (23.5 %) slow-release oral morphine. Normal or intermediate flora was found in 72/132 OMT women (54.5 %) and 2865/3631 controls [78.9 %; OR 0.49 (95 % CI, 0.33–0.71); *p* < 0.001]. Candidiasis occurred more frequently in OMT women than in controls [OR 2.11 (95 % CI, 1.26–3.27); *p* < 0.001]. Findings were inconclusive regarding bacterial vaginosis (± candidiasis) and trichomoniasis. Compared to infants of the control group, those of women with OMT had a lower mean birth weight [MD −165.3 g (95 % CI, −283.6 to −46.9); *p* = 0.006].

**Conclusions:**

Pregnant women with OMT are at risk for asymptomatic vaginal infections. As recurrent candidiasis is associated with preterm delivery, the vulnerability of this patient population should lead to consequent antenatal infection screening at early gestation.

**Electronic supplementary material:**

The online version of this article (doi:10.1186/s12884-016-1003-z) contains supplementary material, which is available to authorized users.

## Background

Opioid dependence during pregnancy is a growing concern, as one-third of the patients entering opioid maintenance therapy (OMT) are women of childbearing age [1]. Oral methadone has been recommended as an OMT for women with opioid dependence during pregnancy since the 1970s and is still considered the standard treatment for this patient population [2]. As an alternative with proven safety and efficacy for pregnant women and their foetuses, buprenorphine was introduced in the early 1990s [3]. The rationale for OMT is to prevent complications of illicit drug use and narcotic withdrawal, as well as to encourage antenatal care and reduce criminal activity [2]. Despite multidisciplinary care, opioid-maintained pregnant women still have an increased risk for adverse pregnancy outcomes such as preterm delivery (PTD), abruption of the placenta, foetal growth restriction, and intrauterine foetal death [4].

In view of the fact that many women with opioid dependence suffer from socioeconomic deprivation, with frequent exposure to violent environments, physical and sexual abuse, the risk for vaginal infections should be considered further [5]. This issue is of particular importance, since vaginal infections at early gestation have been shown to contribute to the multifactorial mechanisms of PTD [6, 7]. Recently, our study group suggested an improvement of obstetrical outcomes through routine screening and consequent treatment for asymptomatic vaginal infections in an overall population of pregnant women at our tertiary referral centre [8].

In addition to the mental stability that is needed, somatic factors should be considered during the antenatal care of pregnant women with OMT. However, no population-based study has yet quantified the likelihood of vaginal infections in these women, accounting for the effect on pregnancy outcomes. Given the known impact of vaginal infections and the possibility of an accumulation of risk factors through OMT and vaginal infections, we considered it a matter of particular interest to investigate the vaginal flora of opioid-maintained pregnant women. Therefore, the present study aimed to stratify the risk of vaginal infections in pregnant women with OMT through analysis of its prevalence in conjunction with obstetrical outcomes.

## Methods

### Setting

We retrospectively analysed data from all women who presented with singleton pregnancies at the Medical University of Vienna, Department of Obstetrics and Gynaecology, between 1 January 2005 and 1 January 2015.

Our centre is specialised in high-risk pregnancy care and serves about 3000 deliveries of publicly health-insured women per year, including referrals from throughout Central Eastern Europe. As part of our routine antenatal service, all women who registered for a planned delivery at our department underwent screening for asymptomatic vaginal infections during a prenatal consultation between 10 + 0 (10 weeks plus 0 days) and 16 + 0 (16 weeks plus 0 days) gestational weeks. According to the official Austrian welfare programme, further obstetric consultations were performed at predetermined time points in obstetric offices [9]. For opioid-dependent, opioid-maintained women, alternating consultations were performed weekly up to daily at the addiction clinic of the Medical University of Vienna. All women were part of a comprehensive, multidisciplinary treatment approach for the management of substance-dependant pregnant women at our tertiary referral centre [10]. Their treatment team consisted of medical doctors, psychologists, nurses, and social workers, who closely collaborated with the Departments of Psychiatry and Psychotherapy, and Pediatrics and Adolescent Medicine, as well as with other hospitals and relevant institutions (e.g., child welfare services, criminal justice system, etc.).

Women on OMT received either a) methadone, b) buprenorphine, or c) slow-release oral morphine (SROM) during pregnancy. Treatment decisions, including OMT choice, dosing, and frequency of visits were individualised and determined by the patient and the treating physicians, following evidence-based treatment recommendations [11]. Alcohol abuse was defined as the regular consumption of alcohol-containing beverages during pregnancy; smoking status was evaluated by the number of cigarettes, with both obtained by questionnaires.

### Procedure

In all women, vaginal smears were obtained by vaginal fluid collection with sterile swabs from the lateral vaginal wall and posterior fornix vaginae. Smears were Gram-stained and microscopically analysed by one of four biomedical laboratory assistants, trained and experienced in gynaecological cytopathology at a laboratory certified according to DIN EN ISO 9001:2008. The protocol involved classification of the vaginal flora as described by Nugent et al. [12]. According to the scoring system, a score of 0–3 was regarded as normal flora, 4–6 as intermediate flora, and 7–10 as bacterial vaginosis (BV). In addition, the presence or absence of *Candida* species (spp.), *Gardnerella* and *Trichomonas vaginalis (T. vaginalis)* was assessed using a microbial identification test with DNA probe technology (BD Affirm™ VP III; Becton Dickinson Co., Sparks, MD, USA). The test uses DNA sequences that bind or hybridize only with the nucleic acid of targeted organisms. The hybridization reaction is highly sensitive and specific in the simultaneous detection and identification of the three major causes of vaginitis. In cases of normal or intermediate flora, women did not receive any treatment. The treatment of BV included clindamycin 2 % vaginal cream for 6 days in cases of a primary infection, oral clindamycin 0.3 g twice daily for 7 days in cases of recurrent BV infection, local clotrimazole 0.1 g for 6 days in cases of vaginal candidiasis, and local metronidazole 0.5 g for 7 days in cases of trichomoniasis [13]. Antibiotic treatment was followed by vaginally applied *Lactobacillus* spp. for 6 days to rebuild the physiological flora [14].

### Study groups

One hundred thirty-two consecutive pregnant women who underwent routine antenatal infection screening were identified for assignment to the OMT group based on their participation in the treatment programme for opioid-dependent women at the addiction clinic. Women of the control group reported that they were neither taking drugs, nor receiving OMT, as evaluated by self-report. There was no significant difference in gestational age at vaginal screening between the study groups. Women who were antenatally referred from other hospitals due to imminent PTD, as well as those who did not undergo the antenatal screening programme, were not eligible for the study. We conducted a matched-group analysis to assess the impact of OMT and opioid dependence on the observed outcome measures. Cases and controls were matched according to the following parameters: maternal age (years), ethnicity (Caucasian vs. non-Caucasian), parity (primipara vs. multipara), previous PTD (yes vs. no), educational level (tertiary vs. non-tertiary), and smoking status (smoking vs. non-smoking). These matching parameters were selected due to their influence on PTD [15–17]. We included only those combinations of the matching variables for which cases and controls were present.

### Outcome measures

The vaginal flora at antenatal screening served as the primary outcome variable, recorded as normal flora, intermediate flora, or vaginal infection in cases of BV and/or colonization with *Candida* spp. and/or *T. vaginalis*. By definition, women with asymptomatic vaginal infections did not have any signs of conspicuous redness, discharge, or vaginal itch. The secondary outcome variables included gestational age at delivery and neonatal birth weight. PTD was defined as spontaneous delivery at or less than 36 + 6 gestational weeks (36 weeks plus 6 days) due to preterm premature rupture of the membranes and/or preterm labour. Stillbirth was defined as the term or preterm delivery of an infant who had died in utero and was born with an Apgar score of 0/0/0. Data were extracted from obstetric databases, patient charts, and microbiologic reports.

### Statistical analysis

Descriptive statistics were used to summarise demographic information. Continuous data are given as mean ± standard deviation (SD), unless stated otherwise. Discrete data are presented as numbers (percentages). For continuous outcomes, a linear mixed model was used, where maternal opioid dependence was a fixed effect, and matched groups were incorporated as a random intercept. Dichotomous outcomes were analysed by means of a conditional logistic regression model that adjusted for all potentially confounding factors. A two-sided *p*-value < 0.05 was considered statistically significant. We accounted for multiplicity by applying the Bonferroni correction to the resulting *p*-values when appropriate. Patient charts were electronically reviewed using PIA Fetal Database, version 5.6.16.917 (General Electric Company, GE Viewpoint, Munich, Germany). Calculations were performed using R-Project for Statistical Computing, version 3.1.3 (R Development Core Team, MA, USA) and SPSS Statistics, version 23.0 (IBM, NY, USA). Figures were constructed using Microsoft Excel, version 14.6.1 (Microsoft, WA, USA).

## Results

A total of 3763 women with singleton pregnancies, who underwent antenatal screening for asymptomatic vaginal infections, were eligible for study inclusion. From this group, we identified 132/3763 (3.5 %) women with OMT (cases) and 3631/3763 (96.5 %) matched controls. In the OMT group, 62/132 (47 %) women received methadone, 39/132 (29.5 %) buprenorphine, and 31/132 (23.5 %) SROM. The mean daily doses of methadone, buprenorphine, and SROM at delivery were 57 ± 29 mg, 6 ± 5 mg, and 410 ± 201 mg, respectively. Patient characteristics of the study participants are shown in Table 1.

**Table 1:** Patient characteristics of the 3763 study participants

Variable	OMT group	Control group	Total
	Mean ± SD	Mean ± SD	Mean ± SD
	*N* (%)	*N* (%)	*N* (%)
Participants	132/3763 (3.5)	3631/3763 (96.5)	3763/3763 (100)
Age at delivery (years)	27.0 ± 4.3	30.4 ± 5.5	30.3 ± 5.5
Caucasian ethnicity	130/132 (98.5)	3588/3631 (98.8)	3718/3763 (98.8)
Parity			
Primiparae	64/132 (48.5)	1186/3631 (32.7)	1250/3763 (33.2)
Multiparae	68/132 (51.5)	2445/3631 (67.3)	2513/3763 (66.8)
Previous PTD	3/68 (4.4)	122/2445 (4.9)	125/2513 (4.9)
Tertiary education	0/132 (0)	347/3631 (9.6)	347/3763 (9.2)
Hepatitis B positive^a^	27/132 (20.5)	n/a	n/a
Hepatitis C positive^a^	36/132 (27.3)	n/a	n/a
HIV positive^a^	8/132 (6)	n/a	n/a
Alcohol abuse	3/132 (2.3)	0/3631 (0)	3/3763 (0.1)
Smoking status			
Smoking	112/132 (84.8)	733/3631 (20.2)	845/3763 (22.5)
Non-smoking	20/132 (15.2)	2898/3631 (79.8)	2918/3763 (77.5)
Cigarettes (per day)	9.9 ± 7.0	1.4 ± 3.9	1.7 ± 4.3

*N* number, *SD* standard deviation, *PTD* preterm delivery, *n/a* not available

^a^serologic testing (HBsAg, anti-HCV, anti-HIV), genotype and viral load, confirmed by PCR analysis

**Fig. 1:**
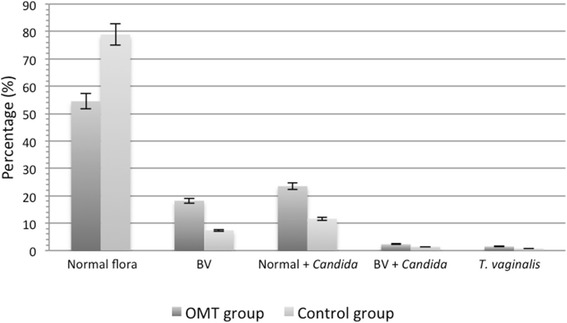
Vaginal flora of the 3763 study participants

On antenatal screening smears, 60/132 (45.5 %) women in the OMT group had an asymptomatic vaginal infection, compared to 766/3631 (21.1 %) controls. The conditional logistic regression model revealed a statistically significant difference in the occurrence of asymptomatic vaginal infections between the study groups [45.5 versus 21.1 %; OR 1.73 (95 % CI, 1.19 to 2.53); *p* = 0.004]. The prevalence of pathogens in the vaginal flora is shown in Fig. 1. Rates of *Candida* colonization were higher in the OMT group compared to the control group [27.3 versus 12.9 %; OR 2.11 (95 % CI, 1.36 to 3.27); *p* < 0.001]. BV rates were also higher in the OMT group compared to the control group, but they were inconclusive as to whether or not a difference was present [18.2 versus 7.4 %; OR 1.53 (95 % CI, 0.93 to 2.53); *p* = 0.094]. No significant differences were found for trichomoniasis. Detailed findings on vaginal screening smears of the 3763 study participants are presented in Table 2.

**Table 2:** Vaginal flora of the 3763 study participants (conditional logistic regression model)

Variable	OMT group	Control group	Total	Odds ratio^a^ (95 % CI)	*p*-value
	*N* (%)	*N* (%)	*N* (%)		
Normal or intermediate flora	72/132 (54.5)	2865/3631 (78.9)	2937/3763 (78)	0.49 (0.33 to 0.71)	<0.001
Vaginal infection	60/132 (45.5)	766/3631 (21.1)	826/3763 (22)	1.73 (1.19 to 2.53)	0.004
Bacterial vaginosis	24/132 (18.2)	269/3631 (7.4)	293/3763 (7.8)	1.53 (0.93 to 2.53)	0.094
Normal or intermediate flora + *Candida* spp.	31/132 (23.5)	420/3631 (11.6)	451/3763 (12)	1.92 (1.21 to 3.05)	0.005
Bacterial vaginosis + *Candida* spp.	3/132 (2.3)	50/3631 (1.4)	53/3763 (1.4)	1.71 (0.47 to 6.24)	0.42
*Trichomonas vaginalis* ^b^	2/132 (1.5)	27/3631 (0.7)	29/3763 (0.8)	1.34 (0.28 to 6.3)	0.712
Involvement of *Candida* spp.	36/132 (27.3)	472/3631 (12.9)	508/3763 (13.5)	2.11 (1.36 to 3.27)	<0.001

*N* number, *CI* confidence interval

^a^control group = reference

^b^incl. 2 cases with T. vaginalis + Candida in each group

Analysis of obstetrical outcomes showed that 19/132 (14.4 %) women in the OMT group and 365/3631 (10 %) women in the control group experienced PTD. Mean gestational age at delivery was 38.6 ± 2.5 weeks in the OMT group and 38.7 ± 2.7 weeks in the control group. Mean birth weight was 2946 ± 550 g and 3245 ± 656 g in the OMT and control groups, respectively. In the OMT group, 2/19 (10.5 %) of the PTD infants had a birth weight of 500–999 g and 17/19 (89.5 %) had a birth weight of 1500–2499 g. As shown in Table 3, 21/365 (5.8 %) of the PTD infants in the control group had a birth weight <500 g, whereas 44/365 (12 %), 30/365 (8.2 %), 215/365 (58.9 %), and 55/365 (15.1 %) had a birth weight of 500–999 g, 1000–1499 g, 1500–2499 g, and ≥2500 g, respectively. In the linear mixed model, birth weight [mean difference, MD, −165.3 g (95 % confidence interval, CI, −283.6 to −46.9); *p* = 0.006] and infant length [MD −0.87 g (95 % CI, −1.58 to −0.17); *p* = 0.015] significantly differed between the study groups. Findings regarding gestational age, Apgar score, umbilical cord arterial pH, and head circumference were inconclusive regarding a possible difference (Table 4).

**Table 3:** Obstetrical outcomes of the 3763 study participants (descriptive statistics)

Variable		OMT group	Control group	Total
		*N* (%)	*N* (%)	*N* (%)
Pregnancy outcome	Live birth	131/132 (99.2)	3614/3631 (99.5)	3745/3763 (99.5)
	Stillbirth	1/132 (0.8)	17/3631 (0.5)	18/3763 (0.5)
Prematurity	Preterm delivery	19/132 (14.4)	365/3631 (10)	384/3763 (10.2)
	No preterm delivery	113/132 (85.6)	3266/3631 (90)	3379/3763 (89.8)
Mode of delivery	Vaginal delivery^a^	68/132 (51.5)	1939/3631 (53.4)	2007/3763 (53.3)
	Caesarean section	53/132 (40.2)	1510/3631 (41.6)	1563/3763 (41.5)
	Instrumental delivery	11/132 (8.3)	182/3631 (5)	193/3763 (5.1)
Gestational week at delivery	<23w 0d	0/132 (0)	30/3631 (0.8)	30/3763 (0.8)
	23w 0d–27w 6d	2/132 (1.5)	38/3631 (1)	40/3763 (1.1)
	28w 0d–31w 6d	2/132 (1.5)	37/3631 (1)	39/3763 (1)
	32w 0d–36w 6d	15/132 (11.4)	260/3631 (7.2)	275/3763 (7.3)
	≥37w 0d	113/132 (85.6)	3266/3631 (90)	3379/3763 (89.8)
Birth weight^b^	<500 g	0/132 (0)	21/3631 (0.6)	21/3763 (0.6)
	500–999 g	2/132 (1.5)	44/3631 (1.2)	46/3763 (1.2)
	1000–1499 g	0/132 (0)	30/3631 (0.8)	30/3763 (0.8)
	1500–2499 g	18/132 (13.6)	215/3631 (5.9)	233/3763 (6.2)
	≥2500 g	112/132 (84.9)	3317/3631 (91.4)	3429/3763 (91.1)

*N* number

^a^incl. vaginal breech delivery

^b^no birth weight available for 4 infants (0.1 %) of the control group

**Table 4:** Obstetrical outcomes of the 3763 study participants (linear mixed model)

Variable	OMT group	Control group	Total	Mean difference^a^ (95 % CI)	*p*-value
	Mean ± SD	Mean ± SD	Mean ± SD		
Birth weight (grams)	2946 ± 550	3245 ± 656	3234 ± 655	−165.3 (−283.6 to −46.9)	0.006
Gestational age (weeks)	38.6 ± 2.5	38.7 ± 2.7	38.7 ± 2.7	−0.05 (−0.45 to 0.54)	0.849
Apgar score					
Apgar at 1 minute	8.5 ± 1.2	8.6 ± 1.1	8.6 ± 1.1	−0.03 (−0.24 to 0.17)	0.743
Apgar at 5 minutes	9.5 ± 1.3	9.6 ± 1.1	9.6 ± 1.1	−0.12 (−0.32 to 0.09)	0.264
Apgar at 10 minutes	9.7 ± 0.9	9.7 ± 1.1	9.7 ± 1.1	−0.04 (−0.23 to 0.16)	0.721
Umbilical cord arterial pH (units)	7.27 ± 0.07	7.27 ± 0.08	7.27 ± 0.08	−0.001 (−0.01 to 0.01)	0.854
Head circumference (cm)	33.6 ± 1.8	34.3 ± 2.1	34.3 ± 2.1	−0.38 (−0.77 to 0.01)	0.054
Length (cm)	49.2 ± 3.0	50.7 ± 3.8	50.7 ± 3.8	−0.87 (−1.58 to −0.17)	0.015

*SD* standard deviation, *CI* confidence interval, *cm* centimetres

^a^control group = reference

## Discussion

In the present study, we found increased odds for asymptomatic vaginal infections and candidiasis in opioid-dependent women receiving OMT. Our findings suggest that there is a potential influence of OMT and maternal opioid dependence on the vaginal flora, which should be taken into account during antenatal care.

Opioid-dependent women commonly suffer from unemployment, co-addicted partners, and intimate partner violence [10, 19]. In contrast to illicit drug abuse, OMT has a normalising effect on endocrinological and immunological functioning, which could lead to unexpected pregnancy in OMT women with insufficient birth control measures [20]. With regard to the socioeconomic situation, promiscuous behaviour might foster health problems including infectious diseases of the lower genital tract. Curry et al. [21] reported that maternal physical and sexual abuse, which is likely associated with substance abuse and the chaotic lifestyles of addicted mothers, was related to poor obstetric histories. Our study confirmed the low socioeconomic and educational status of OMT women, who were young and often tobacco dependent.

Knowing that women with OMT are more likely suffering from socioeconomic deprivation and experiencing adverse pregnancy outcomes, one might postulate a potential cumulative effect in cases of vaginal infection. We found the rate of *Candida* involvement to be significantly higher in the screening smears of OMT women than in those of the control group. Independent from other potentially unknown confounders, we found that, in our population, asymptomatic vaginal infections, and candidiasis in particular, were common findings among pregnant women with OMT. Rates of BV and *T. vaginalis* were also higher in the OMT group, but the differences were not statistically significant (Fig. 1). Reported candidiasis rates of 10–15 % at early gestation are comparable to the prevalence observed in our control group (12.9 %), but not to that in the OMT group (27.3 %) [22–24]. According to our previously published data, there is an association between recurrent candidiasis and PTD, but not between one-time candidiasis and PTD [25]. This finding could be relevant, since post-treatment re-colonization rates with *Candida* spp. are typically high, with rates of up to 50 % within 1 month after completing a short-term antimycotic therapy [26]. In addition, certain anaerobe microorganisms, which are recognized to be associated with BV, PTD and stillbirth, could be part of the vaginal flora, although they are uncultivable and not detectable on Gram-stains [27, 28]. Culture-independent molecular-based techniques could have provided detailed information about the composition of the vaginal flora, probably indicating a far greater diversity of microorganisms, and thereby enhancing the existing knowledge from Gram-stain and culture-dependent techniques [29].

Previous studies reported that women receiving OMT during pregnancy are at risk for PTD, low birth weight, and small head circumference [30, 31]. Our study detected a 4.4 % increase in PTD within the OMT group, which was, however, not significant with regard to the mean gestational age. Peles et al. [32] reported that the best obstetrical outcomes in opioid-maintained women, defined by gestational age and birth weight, were achieved by longer duration of OMT and substance abstinence, which emphasises the importance of OMT stabilisation before and during pregnancy. Moreover, co-medication and polydrug abuse might be associated with reduced foetal growth and adverse outcomes [33, 34]. Dryden et al. [35] reported that 23 % of the infants born to OMT mothers weighed less than the 9th percentile at delivery. In our study, we found indicators for impaired foetal growth, such as low birth weight and short length, in infants of opioid-maintained mothers. Because our groups were matched for maternal characteristics, our results are consistent with those of Mactier et al. [30], who postulated that reduced foetal growth cannot be fully explained by maternal tobacco abuse, age, or parity.

From a clinical point of view, our findings indicate that there is a need for the implementation of routine screening programmes to prevent vaginal infections in OMT women. These women constitute a select patient cohort facing high-risk pregnancies and a special need for early pregnancy care [36]. Cases of women first presenting for care at delivery should certainly be prevented, since the absence of prenatal care allows for an accumulation of risk factors, which in turn increases PTD risk and subsequent costs to society. Comprehensive care for the prevention of PTD should be individualised for every pregnant women. Screening programmes for the prevention of vaginal infections should become part of international guidelines for the prenatal care of OMT women. Although their partners might not obligatorily be screened, the increased risk for human immunodeficiency virus (HIV) infection and hepatitis should also be considered.

To the best of our knowledge, this paper is the first to evaluate the vaginal flora of pregnant women with OMT. Beyond the aim of our study, which was to evaluate their vaginal flora, we considered it essential to report our obstetrical outcomes. Indeed, our study has several limitations, including the retrospective design, the case-control setting, and the lack of patient characteristics in the control group. We are aware that characteristics might differ between opioid-dependent and non-opioid-dependent women. Although we matched cases and controls for potentially confounding factors, we were unable to adjust for HIV infection and hepatitis. There might be an increased prevalence of these infections among women with an abnormal vaginal flora, but it remains unclear whether this difference is related to the infection, the socioeconomic deprivation, the immunodeficiency, or to other, unknown factors [37, 38]. Moreover, the increased susceptibility for vaginal infections in OMT women might also be induced by a frequent change of sexual partners, high sexual activity, underlying diseases, or co-abuse of other drugs that we were unable to adjust for [39]. Finally, it remains unclear to what extent the choice and dosage of OMT had an effect on the outcomes of our study.

## Conclusion

In conclusion, our data demonstrate that pregnant women with OMT are at risk for asymptomatic vaginal infections at early gestation. In particular, *Candida* spp. is more frequently involved in vaginal screening smears of women with OMT, compared to those of the overall pregnant cohort. Considering the known effect of vaginal infections on obstetrical outcomes, our findings indicate the need for a multidisciplinary approach in the care of pregnant opioid-dependent, opioid-maintained women, including comprehensive screening and treatment for vaginal infections, in order to potentially prevent the accumulation of risk factors for adverse perinatal outcomes and PTD. To confirm our findings, prospective studies are warranted that include the evaluation of the vaginal flora by culture or PCR.

## Abbreviations

BV, bacterial vaginosis; *Candida* spp., *Candida* species; MD, mean difference; OMT, opioid maintenance therapy; OR, odds ratio; PTD, preterm delivery; SD, standard deviation; SROM, slow-release oral morphine

## Additional file

Additional file 1: Dataset supporting the conclusions of this article. (XLS 2634 kb)

## References

[1] Johnson RE, Jones HE, Fischer G (2003). Use of buprenorphine in pregnancy: patient management and effects on the neonate. Drug Alcohol Depend.

[2] ACOG (2012). Committee opinion No. 524: opioid abuse, dependence, and addiction in pregnancy. Obstet Gynecol.

[3] Jones HE, Kaltenbach K, Heil SH, Stine SM, Coyle MG, Arria AM, O’Grady KE, Selby P, Martin PR, Fischer G (2010). Neonatal abstinence syndrome after methadone or buprenorphine exposure. N Engl J Med.

[4] Brogly SB, Saia KA, Walley AY, Du HM, Sebastiani P (2014). Prenatal buprenorphine versus methadone exposure and neonatal outcomes: systematic review and meta-analysis. Am J Epidemiol.

[5] Illangasekare SL, Burke JG, Chander G, Gielen AC (2014). Depression and social support among women living with the substance abuse, violence, and HIV/AIDS syndemic: a qualitative exploration. Womens Health Issues.

[6] Romero R, Espinoza J, Kusanovic JP, Gotsch F, Hassan S, Erez O, Chaiworapongsa T, Mazor M (2006). The preterm parturition syndrome. BJOG.

[7] Jones HE, Finnegan LP, Kaltenbach K (2012). Methadone and buprenorphine for the management of opioid dependence in pregnancy. Drugs.

[8] Farr A, Kiss H, Hagmann M, Marschalek J, Husslein P, Petricevic L (2015). Routine use of an antenatal infection screen-and-treat program to prevent preterm birth: long-term experience at a tertiary referral center. Birth.

[9] Sperno R, Rudelstorfer R, Gruber W (1985). Effect of prenatal care in general practice and in the clinic on the course of pregnancy and labor. Wien Med Wochenschr.

[10] Winklbaur B, Kopf N, Ebner N, Jung E, Thau K, Fischer G (2008). Treating pregnant women dependent on opioids is not the same as treating pregnancy and opioid dependence: a knowledge synthesis for better treatment for women and neonates. Addiction.

[11] Unger A, Jagsch R, Jones H, Arria A, Leitich H, Rohrmeister K, Aschauer C, Winklbaur B, Bawert A, Fischer G (2011). Randomized controlled trials in pregnancy: scientific and ethical aspects. Exposure to different opioid medications during pregnancy in an intra-individual comparison. Addiction.

[12] Hillier SL, Krohn MA, Nugent RP, Gibbs RS (1992). Characteristics of three vaginal flora patterns assessed by gram stain among pregnant women. Vaginal infections and prematurity study group. Am J Obstet Gynecol.

[13] Lamont RF, Nhan-Chang CL, Sobel JD, Workowski K, Conde-Agudelo A, Romero R (2011). Treatment of abnormal vaginal flora in early pregnancy with clindamycin for the prevention of spontaneous preterm birth: a systematic review and metaanalysis. Am J Obstet Gynecol.

[14] Petricevic L, Witt A (2008). The role of Lactobacillus casei rhamnosus Lcr35 in restoring the normal vaginal flora after antibiotic treatment of bacterial vaginosis. BJOG.

[15] Goldenberg RL, Culhane JF, Iams JD, Romero R (2008). Epidemiology and causes of preterm birth. Lancet.

[16] Alnaif B, Drutz HP (2001). The association of smoking with vaginal flora, urinary tract infection, pelvic floor prolapse, and post-void residual volumes. J Low Genit Tract Dis.

[17] Wasiela M, Hanke W, Kalinka J (2001). Association between abnormal microbiological flora of the lower genital tract in early pregnancy and socio-economic, demographic and environmental risk factors. Med Sci Monit.

[18] Vandenbroucke JP, von Elm E, Altman DG, Gotzsche PC, Mulrow CD, Pocock SJ, Poole C, Schlesselman JJ, Egger M, STROBE initiative (2007). Strengthening the reporting of observational studies in epidemiology (STROBE): explanation and elaboration. Ann Intern Med.

[19] Moore BC, Easton CJ, McMahon TJ (2011). Drug abuse and intimate partner violence: a comparative study of opioid-dependent fathers. Am J Orthopsychiatry.

[20] Kreek MJ, Schluger J, Borg L, Gunduz M, Ho A (1999). Dynorphin A1-13 causes elevation of serum levels of prolactin through an opioid receptor mechanism in humans: gender differences and implications for modulation of dopaminergic tone in the treatment of addictions. J Pharmacol Exp Ther.

[21] Curry MA, Perrin N, Wall E (1998). Effects of abuse on maternal complications and birth weight in adult and adolescent women. Obstet Gynecol.

[22] Cotch MF, Hillier SL, Gibbs RS, Eschenbach DA (1998). Epidemiology and outcomes associated with moderate to heavy Candida colonization during pregnancy. Vaginal Infections and Prematurity Study Group. Am J Obstet Gynecol.

[23] Krauss-Silva L, Almada-Horta A, Alves MB, Camacho KG, Moreira ME, Braga A (2014). Basic vaginal pH, bacterial vaginosis and aerobic vaginitis: prevalence in early pregnancy and risk of spontaneous preterm delivery, a prospective study in a low socioeconomic and multiethnic South American population. BMC Pregnancy Childbirth.

[24] Akinbiyi AA, Watson R, Feyi-Waboso P (2008). Prevalence of Candida albicans and bacterial vaginosis in asymptomatic pregnant women in South Yorkshire, United Kingdom. Outcome of a prospective study. Arch Gynecol Obstet.

[25] Farr A, Kiss H, Holzer I, Husslein P, Hagmann M, Petricevic L (2015). Effect of asymptomatic vaginal colonization with Candida albicans on pregnancy outcome. Acta Obstet Gynecol Scand.

[26] Fleury FJ (1981). Adult vaginitis. Clin Obstet Gynecol.

[27] Han YW, Fardini Y, Chen C, Iacampo KG, Peraino VA, Shamonki JM, Redline RW (2010). Term stillbirth caused by oral Fusobacterium nucleatum. Obstet Gynecol.

[28] Bretelle F, Rozenberg P, Pascal A, Favre R, Bohec C, Loundou A, Senat MV, Aissi G, Lesavre N, Brunet J (2015). High Atopobium vaginae and Gardnerella vaginalis vaginal loads are associated with preterm birth. Clin Infect Dis.

[29] Lamont RF, Sobel JD, Akins RA, Hassan SS, Chaiworapongsa T, Kusanovic JP, Romero R (2011). The vaginal microbiome: new information about genital tract flora using molecular based techniques. BJOG.

[30] Mactier H, Shipton D, Dryden C, Tappin DM (2014). Reduced fetal growth in methadone-maintained pregnancies is not fully explained by smoking or socio-economic deprivation. Addiction.

[31] Greig E, Ash A, Douiri A (2012). Maternal and neonatal outcomes following methadone substitution during pregnancy. Arch Gynecol Obstet.

[32] Peles E, Schreiber S, Bloch M, Dollberg S, Adelson M (2012). Duration of methadone maintenance treatment during pregnancy and pregnancy outcome parameters in women with opiate addiction. J Addict Med.

[33] Brown HL, Britton KA, Mahaffey D, Brizendine E, Hiett AK, Turnquest MA (1998). Methadone maintenance in pregnancy: a reappraisal. Am J Obstet Gynecol.

[34] Lund IO, Skurtveit S, Engeland A, Furu K, Ravndal E, Handal M (2013). Prescription drug use among pregnant women in opioid maintenance treatment. Addiction.

[35] Dryden C, Young D, Hepburn M, Mactier H (2009). Maternal methadone use in pregnancy: factors associated with the development of neonatal abstinence syndrome and implications for healthcare resources. BJOG.

[36] Metz VE, Comer SD, Wuerzl J, Pribasnig A, Fischer G (2014). Characteristics and quality of life of opioid-dependent pregnant women in Austria. Arch Womens Ment Health.

[37] Vallone C, Rigon G, Lucantoni V, Putignani L, Signore F (2012). Pregnancy in HIV-positive patients: effects on vaginal flora. Infect Dis Obstet Gynecol.

[38] Warren D, Klein RS, Sobel J, Kieke B, Brown W, Schuman P, Anderson J, Cu-Uvin S, Mayer K, Jamieson DJ (2001). A multicenter study of bacterial vaginosis in women with or at risk for human immunodeficiency virus infection. Infect Dis Obstet Gynecol.

[39] Fethers K, Twin J, Fairley CK, Fowkes FJ, Garland SM, Fehler G, Morton AM, Hocking JS, Tabrizi SN, Bradshaw CS (2012). Bacterial vaginosis (BV) candidate bacteria: associations with BV and behavioural practices in sexually-experienced and inexperienced women. PLoS One.

